# Computed Tomography and Ultrasounds for the Follow-up of Hepatocellular Carcinoma Ablation: What You Need to Know

**DOI:** 10.3390/diagnostics6010009

**Published:** 2016-02-04

**Authors:** Alexios Kelekis, Dimitrios Filippiadis

**Affiliations:** 2nd Radiology Department, University General Hospital “ATTIKON”, 1 Rimini str, 12462 Athens, Greece; dfilippiadis@yahoo.gr

**Keywords:** liver, tumor, ablation, imaging, follow-up

## Abstract

Image-guided tumor ablation provides curative treatment in properly selected patients or appropriate therapeutic options whenever surgical techniques are precluded. Tumor response assessment post ablation is important in determining treatment success and future therapy. Accurate interpretation of post-ablation imaging findings is crucial for therapeutic and follow-up strategies. Computed Tomography (CT) and Ultrasound (US) play important roles in patients’ follow-up post liver thermal ablation therapies. Contrast-enhanced ultrasound (CEUS) can provide valuable information on the ablation effects faster and at a lower cost than computed tomography or magnetic resonance imaging. However, a disadvantage is that the technique cannot examine total liver parenchyma for disease progression as CT and Magnetic Resonance (MR) imaging can. Follow-up strategies for assessment of tumor response includes contrast enhanced multiphasic (non-contrast, arterial, portal, delayed phases) imaging with Computed Tomography at three, six, and 12 months post ablation session and annually ever since in order to prove sustained effectiveness of the ablation or detect progression.

## 1. Introduction

Hepatocellular carcinoma is the fifth most common cancer type, accounting for 7% of all cancers and the third most common cause of cancer-related death [1,2]. Imaging-guided ablation provides curative treatment in properly selected patients or appropriate therapeutic options whenever surgical techniques are precluded [2]. Concerning hepatocellular carcinoma, the two most commonly applied heat-based ablation techniques are radiofrequency (RFA) and microwave (MWA) ablation. During MWA, there is more efficient transfer of heat and subsequent faster tissue heating when compared with radiofrequencies. In contrast to radiofrequency ablation, where the energy circulation is hindered by high tissue impedance, with microwave, penetration and thus heating of any tissue can be performed. Furthermore, microwaves are confined close to the antenna as opposed to radiofrequency which during ablation flows through the body to reach the grounding pads. Microwaves are governed by higher heating efficiency than radiofrequency which renders them unaffected by “heat sink” effect and blood vessels resulting in larger ablation volumes achieved in less time. Alternatives in our therapeutic armamentarium include cryoablation, irreversible electroporation, light activated drug therapy and laser induced thermotherapy (LITT) [3,4,5,6].

Imaging in ablation practice apart from acting as a guiding mode is also used for evaluation of the ablation zone (*i.e.*, treatment success), for follow-up and as a proof for need or not of additional therapy. Ideally, imaging post ablation should be performed by a reproducible and repeatable, cost-effective method that carries no risk to the patient and which can detect residual viable tumor tissue or confirm complete tumor necrosis. Since tumor marker levels can be normal even in cases of partial necrosis, the evaluation of ablation’s effect is based mainly upon imaging studies [7]. Illustration of incomplete necrosis is of paramount importance in order to retreat the patient.

The purpose of this paper is to describe state-of-the art imaging post liver thermal ablation focusing on ultrasound and computed tomography, to analyze optimal imaging timing in relation to therapeutic and follow-up strategies, and to illustrate future imaging potential and developments.

## 2. Assessment of Tumor Response

Assessing the outcome of ablation can be performed either by morphological (World Health Organization (WHO), Response Evaluation Criteria in Solid Tumors (RECIST) 1.0 and 1.1) or by functional (EASL, mRECIST) criteria [1,2,6,7]. Magnetic Resonance Imaging (MRI) with diffusion-weighted sequences and perfusion either in a Computed Tomography (CT) or MRI evaluate tumor response with functional criteria. Morphological criteria evaluating the size of a tumor post ablation cannot achieve proper assessment as opposed to functional criteria that provide significant details about the imaging behavior of Hepatocellular carcinoma (HCC) post liver ablation.

By definition, the term curative ablation describes an ablation zone, which covers the whole tumor along with a clear margin (5–10 mm of normal liver parenchyma) all around the tumor [8,9,10]. Incomplete ablation refers to the presence of tumor remnant at the first post-ablation scan [8,9,10]. Local tumor progression or tumor recurrence refers to tumor development within an ablation zone or at a maximum distance of 1cm around it in a patient whose follow-up imaging had already shown curative ablation [8,9,10].

**Figure 1:**
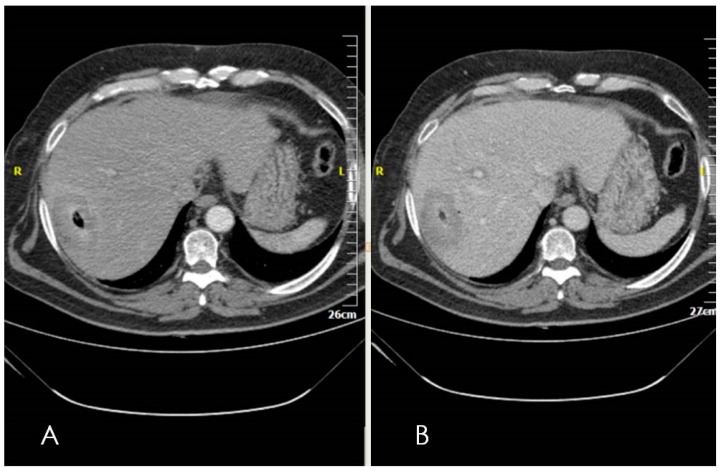
Computed Tomography axial scan in arterial (**A**) and portal venous phase; (**B**) immediately post ablation illustrating lack of complications and signs of successful ablation including no contrast enhancement within the ablation zone. Gas formation is seen in the center of the ablation zone demarcating the location of the microwave probe.

Immediately post ablation session, a CT scan seems mandatory (Figure 1). Multiphase scan (including non-contrast, arterial and portal phase) of the upper abdomen (from lung base to right kidney level) should be performed in order to identify any potential complications (e.g., pneumothorax, hemorrhage, *etc.*) but to preliminarily evaluate therapeutic outcomes as well. Ablation outcome is evaluated within six weeks post treatment in order for post ablative inflammatory changes to subside. This first post-ablation scan serves as the new baseline scan for future evaluation. Subsequently, follow-up strategy for assessment of tumor response includes contrast enhanced multiphasic (non-contrast, arterial, portal, delayed phases) imaging with Computed Tomography at three, six, and 12 months post ablation session and annually ever since in order to prove sustained effectiveness of the ablation or detect progression.

Evaluation of the ablation outcome is important for determining treatment success and future therapy. WHO criteria and RECIST included the initial guidelines for tumor response assessment [11,12]. Both WHO and RECIST criteria appreciate tumor response on the basis of determining changes in tumor and its anatomic size therefore they address tumor response on the basis of tumor shrinkage [11,12]. By definition, a successful ablation zone should be larger than the tumor since it includes the safety margin as well. In addition, tumor recurrence is often illustrated as an enhanced nodule within the boundaries of the ablation zone with no resulting size changes. Therefore, tumor response criteria based upon changes in tumor size seem to be lacking the necessary sensitivity for the assessment of post-ablative results.

Nowadays, mRECIST criteria are used for tumor response assessment requiring image acquisition protocols with contrast medium, as well as optimization and consistency in the same protocol throughout follow-up examinations [13,14]. Assessment of overall response with mRECIST criteria includes evaluation of the response of both target and non-target lesions as well as potential new lesions. However, concerning the assessment of target lesion's response, mRECIST appreciate apart from size reduction and the intra-tumoral arterial enhancement as viable tumor tissue [13,14].

## 3. Ultrasounds

**Figure 2:**
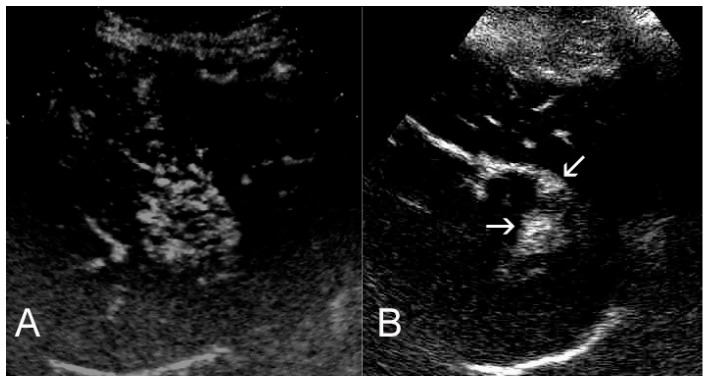
CEUS diagnosis of partial response post ablation. CEUS images of a hypervascular HCC at baseline (A), and one month post ablation (B). At baseline, the tumor shows intense, early enhancement. Post ablation, a nodular residual tumor, also exhibiting strong, early enhancement, is noted (arrows).

Standard ultrasound structural changes such as hyperechogenicity post treatment are not reliable for the evaluation of ablation’s outcome since residual viable tumor and coagulation necrosis areas are not adequately demonstrated [7]. Contrast-enhanced ultrasound (CEUS) can provide valuable information and assess tumor response faster and at lower cost than computed tomography or magnetic resonance imaging. Re-injection of the contrast medium at the late or post vascular phase will differentially diagnose remnant/recurrence or not in a suspicious lesion (Figure 2). One disadvantage is that CEUS cannot be used in order to examine the total liver parenchyma and the rest of the abdomen for intra- and extra-hepatic disease progression as CT and MR imaging can. Recently a joint World Federation of Ultrasound in Medicine and Biology (WFUMB)-European Federation of Societies for Ultrasound in Medicine and Biology (EFSUMB) venture based upon extended literature survey has resulted in the release of guidelines for the hepatic applications of CEUS [15,16]. Recommended uses of CEUS in the follow-up period include assessment of tumor response whenever contrast enhanced CT or MRI are contraindicated or inconclusive, or in addition to the aforementioned studies [16]. Furthermore, CEUS can be used in the immediate post ablative period to detect residual non-ablated tumor which can immediately be re-treated. With this specific strategy, incomplete ablation rate decreases from 16% to 6% [17]. In addition, the immediate post-ablation CEUS seems comparable on terms of residual disease detection with immediate CT scans [18].

More recent advancements in technology allow demonstration of ablated areas and residual lesions in three dimensions (CE 3D US), this technique is extremely useful for detailed outcome evaluation and seems to be governed by good concordance with the results of contrast enhanced CT in three dimensions [19]. However, it must be noted that both techniques are governed by a limitation in the detection of small or microscopic residual tumors and in the prediction of local re-growth of the treated lesions [20].

## 4. Computed Tomography (CT)

**Figure 3:**
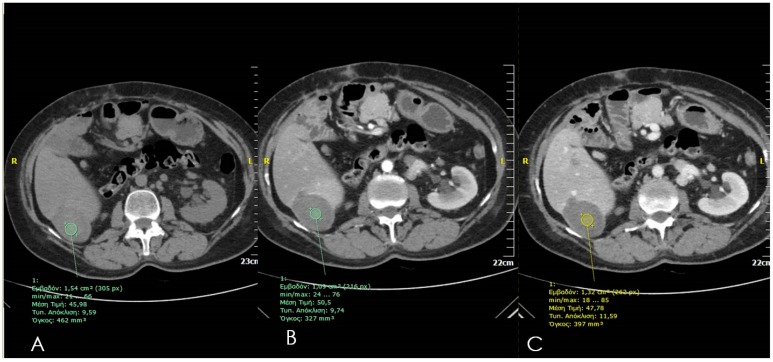
Computed Tomography axial scan prior (A) and post IV injection of contrast medium in arterial and (B) and portal venous phase; (C) one month post ablation illustrating a lack of contrast enhancement within the ablation zone.

Dynamic Contrast enhanced multi-detector CT scans (unenhanced-arterial-portal-delayed phase) are widely used for the evaluation of tumor response in the post-ablation follow-up. Total necrosis and successful ablation of a hepatic lesion appears in CT as a homogeneous non-enhancing attenuation extending all over the ablated volume (Figure 3). A hypoattenuating area failing to enhance in both arterial and portal venous phases is a sign of successful ablation [7]. In the immediate post-session, scan gas can be seen which in most cases resolves until the one month follow-up scan, these gas bubbles are either introduced along the needle’s insertion path or are associated with tissue necrosis. In the early post-ablation period, a central area of high attenuation is illustrated along the needle tract that usually disappears by the next follow-up scan. Occasionally, a thin rim of peripheral enhancement might be seen surrounding the ablation zone, which could represent granulation tissue [21]. Evidence of tumor remnants or recurrence includes residual or new areas of contrast enhancement in arterial or portal venous phase either at the periphery or internally to the ablation zone [21]. Fibrous tissue due to scarring enhances with a slow and persistent mode throughout arterial, portal and delayed phase as opposed to tumor remnants or recurrence which illustrate arterial enhancement and portal/delayed wash-out. Periablational enhancement (uniform, concentric and symmetric with smooth inner margins) may last up to six months and must be differentiated from incomplete local treatment (irregular peripheral enhancement) [8,22]. In cases of local tumor progression follow-up, CT scans illustrate an irregular thick rim of enhancement around the ablation zone, nodules at the periphery of the ablation zone or gross enlargement of the ablated volume [22].

Abnormal findings of imaging after percutaneous ablation of hepatocellular carcinoma apart from residual or recurrent disease include biliary or vascular complications, infection and any other non-target injury [23]. Biliary complications include stricture, fistulas (biliovenous, biliocutaneous, biliopleural), biloma and gallbladder injury or perforation [23]. Vascular complications are caused either by mechanical or thermal injury and include hemorrhage (parenchymal, perihepatic, intraperitoneal), fistula (arteriovenous or arterioportal), pseudoaneurysm or occlusion of the hepatic artery, portal vein thrombosis and hepatic infarction [23].

Computed Tomography scans are governed by lower sensitivity (44% *versus* 89%) for residual disease detection when compared to Magnetic Resonance Imaging [24]. Keeping track of technological evolution, arterial enhancement fraction color seems to predict tumor recurrence earlier than contrast medium enhanced CT [25]. In these techniques, subtraction assists in better illustrating subtle arterial enhancement differences which predict recurrence [25]. In addition, volumetric methods in CT scans might increase sensitivity of the technique for the assessment of tumor response [26]. In these techniques, an automatic or semiautomatic 3D segmentation of the tumor is performed; however, one should keep in mind that segmentation in the liver is extremely difficult due to the variations of size, shape and density of the lesion. Changes in tumor vascularity and perfusion post ablation can be assessed with perfusion CT; arterial enhancement fraction can be illustrated with a color mapping and is used as a surrogate marker for perfusion CT [22,27]. However, it must be noted that the use of such functional imaging for the evaluation of treatment response is impeded by the lack of standardization.

## 5. Conclusions

Specific response criteria based upon certain imaging findings are necessary in order to evaluate the outcome of ablation in hepatocellular carcinoma. Outcome evaluation is crucial not only for valuing a specific ablation technique but also for predicting a patient’s prognosis. In order to successfully evaluate the outcome, post ablation prerequisites include understanding of various response systems, thorough imaging in predefined time points, and occasionally multimodality imaging. Ultrasounds with contrast agents and CTs are widely available and can be considered key players among equals for the evaluation of therapeutic outcomes.
